# 5-Hydroxymethylcytosine Loss in Conjunctival Melanoma

**DOI:** 10.3390/dermatopathology8020023

**Published:** 2021-06-05

**Authors:** Alexandre Stahl, Nicolo Riggi, Katya Nardou, Michael Nicolas, Gurkan Kaya, Alexandre Moulin

**Affiliations:** 1 Jules-Gonin Eye Hospital, Lausanne University, 1004 Lausanne, Switzerland; alexandre.stahl@unil.ch (A.S.); katya.nardou@fa2.ch (K.N.); michael.nicolas@fa2.ch (M.N.); 2 Experimental Pathology, University Hospital of Lausanne (CHUV), 1011 Lausanne, Switzerland; Nicolo.Riggi@unil.ch; 3 Dermatopathology Unit, Dermatology and Clinical Pathology Departments, University Hospitals of Geneva, 1205 Geneva, Switzerland; gkaya@hcuge.ch

**Keywords:** conjunctival melanoma, epigenetics, 5-hydroxymethylcytosine, 5-methylcytosine, TET2

## Abstract

Aims: Conjunctival and cutaneous melanoma partially share similar clinical and molecular backgrounds. As 5-hydroxymethylcytosine (5-hmC) loss has been demonstrated in cutaneous melanoma, we decided to assess if similar changes were occurring in conjunctival melanoma. Methods: 5-methylcytosine (5-mC), 5-hmC and TET2 were respectively identified by immunohistochemistry and RNA ISH in 40 conjunctival nevi and 37 conjunctival melanomas. Clinicopathological correlations were established. Results: 5-mC, TET2 and 5-hmC were respectively identified in 67.5%, 95% and 100% of conjunctival nevi and in 81.1%, 35.1% and 54% of conjunctival melanomas. A significant 5-hmC and TET2 loss was identified in conjunctival melanoma comparing to nevus, as well as a significant correlation between TET2 and 5-hmC expression. In the melanomas, 5-hmC expression was only significantly associated with local lymphatic invasion, but not with other clinicopathological parameters. There was a correlation between TET2 expression and the localization of the tumors. 5-mC expression was not associated with any clinicopathological parameters. Conclusions: We identified a significant 5-hmC loss in conjunctival melanoma similar to cutaneous melanoma. This loss may possibly be attributed to TET2 loss or IDH1 mutations. 5-hmC loss in conjunctival melanoma may help in the differential diagnosis between atypical conjunctival nevus and conjunctival melanoma.

## 1. Introduction

Conjunctival melanoma is a rare malignant tumor, with a 10-year disease-specific mortality ranging from 9% to 35% [1]. The incidence of conjunctival melanoma in Europe and the US is around 0.2–0.7 cases per million and this disease predominantly affects Caucasians and the elderly [2]. The incidence increases in Caucasian populations [3] in Europe [4] and the United States [5,6].

Primary treatment of conjunctival melanoma consists of local surgical excision with wide margins and adjuvant therapy (cryotherapy, brachytherapy, and/or topical application of mitomycin C) [7,8]. Regional and systemic metastatic dissemination occurs in approximately 30% of patients within 3 years [7].

Conjunctival melanoma is believed to arise from the malignant transformation of melanocytes localized either in the bulbar conjunctiva exposed to the sun or in the tarsal or forniceal conjunctiva not exposed to the sun [3]. The majority of conjunctival melanomas develop from preexisting lentiginous atypical melanocytic proliferations (PAM with atypia/C-MIN), while a minority arise from preexisting nevus or de novo [8]. Conjunctival melanoma is biologically very different from uveal melanoma and appears closer to cutaneous melanoma [9]. Recent evidence suggests that cutaneous melanoma and conjunctival melanoma share similar molecular characteristics, notably similar mutations in driver genes such as *BRAF*, *NRAS* and *NF1* [9,10] as well as a UV light signature [9,11].

We have previously demonstrated that this genetic background leads to increased activation of the MAPK signaling pathway, as well as the PI3K/mTOR pathway in conjunctival melanoma [12]. In addition, according to our recent investigations, MAP kinase, Hippo and WNT signaling pathways are frequently altered in conjunctival melanoma [9].

DNA methylation at the 5 position of cytosine (5-mC) is a key epigenetic mark that is critical for various biological and pathological processes. 5-mC can be converted to 5-hydroxymethylcytosine (5-hmC) by the ten–eleven translocation (TET) family of DNA hydroxylases [13]. Lian CG et al. reported that 5-hmC loss is an epigenetic feature of melanoma, with diagnostic and prognostic implications [13]. Genome-wide mapping of 5-hmC revealed a loss of the 5-hmC landscape occurring in the melanoma epigenome. Loss of 5-hmC resulted from TET or isocitrate dehydrogenase 2 (IDH2) downregulation. Inactivating TET mutations or inhibition of TET activity by IDH 1/2 mutations have also previously been reported in tumors with 5-hmC loss [14]. Rebuilding the 5-hmC landscape in melanoma cells by reintroducing active TET2 or IDH2 suppressed melanoma growth and increased tumor-free survival in animal models [13]. This study revealed a critical function of 5-hmC in melanoma development and directly linked the IDH and TET activity-dependent epigenetic pathway to 5-hmC mediated suppression of melanoma progression, suggesting a new strategy for epigenetic cancer therapy [13].

As investigations of epigenetic alterations occurring in conjunctival melanoma have not been thoroughly explored, we assessed in this study the expression of 5-hmC, 5-mC and TET2 in benign and malignant conjunctival melanocytic proliferations.

## 2. Material and Methods

Authorization from the ethics committee (authorization CER-VD 2019-0630) was granted. Tissue samples from patients treated at the Jules-Gonin Eye Hospital (Lausanne, Switzerland) were selected. The study included samples that had been archived from 1998 to 2020. The study samples included 40 cases of benign conjunctival nevi (25 compound nevi and 15 subepithelial nevi) and 37 cases of conjunctival melanomas.

Patient data regarding age at diagnosis, gender, tumor location, recurrence, metastasis, mortality and possible precursor lesions (nevus or PAM) were extracted from the clinical records of Jules-Gonin Eye Hospital. The following histopathological elements were also recorded: diagnosis, mitotic activity, depth of invasion, lymphatic invasion and genetic mutation profile (*BRAF* and *NRAS*) if available, as well as TNM stage.

### 2.1. Immunohistochemistry

The paraffin-embedded and formalin-fixed tissues were recovered from the archives of the pathology laboratory of the Jules-Gonin Eye Hospital. Sections of 4 μm were cut and hematoxylin-eosin staining was performed. The sections were incubated with 5-hmC antibody (Active Motif, 39769, Carlsbad, CA, USA) on an automated Benchmark XT platform (F. Hoffmann-La Roche AG, Basel). After blocking endogenous peroxidase and antigenic unmasking at pH 9, the sections were incubated for one hour with the 5-mC antibody (Active Motif, 39649, clone 33D3, 1/300 dilution). A streptavidin/biotin method with 3,3′-diaminobenzidine tetrachloride (DAB) was used for signal detection (DAKO EnvisionTM + System/HRP Dual Link). After immunohistochemical staining, the slides were counterstained for 10 s in Meyer’s hematoxylin. Two observers (A.S. and A.M.) independently reviewed and noted the immunohistochemistry results. One section per specimen was evaluated. For each cell location, we listed the proportion of stained cells using a scale of 1 to 3 (1 = positive staining in <10% of the cells; 2 = positive staining in 10–50% of the cells; 3 = positive staining in >50% of the cells). A score of 1 was interpreted as negative and scores of 2 and 3 as positive. When the independent assessment of the score differed, the slides were examined jointly to reach a mutual agreement.

For each melanoma case, Ki67 immunoreactivity was determined as the mean of positive cells determined in 5 HPF (400×, 0.115 mm^2^/HPF).

### 2.2. In Situ Hybridization

The detection of TET2 was performed by RNA ISH using RNAscope technology (Advanced Cell Diagnosis Inc., Newark, CA, USA). Preservation of RNA integrity within the material was assessed by expression of the POLR2A control gene (ACD, 310451) and TET2 expression was performed using TET2 probes (ACD, 420051) using the ACD Hyb EZ II hybridization system (ACD, 321720) according to the manufacturer’s recommendations. Negative control was achieved by expression of the bacterial DAP B gene. Cases where POLR2A expression was not preserved were not included in the study. The conjunctival epithelium served as a positive internal control for TET2 expression. Signal detection was revealed with Fast Red dye (ACD, 322360). Signal evaluation was performed by two independent observers (AM and AS) according to the following system: score 1, 1–3 dots/cell; score 2, 4–9 dots/cell; score 3, 10–15 dots/cell; score 4 > 15 dots/cell). Where the independent scoring of the score differed, the slides were examined together to reach a mutual agreement.

### 2.3. Statistical Tests

The statistical analysis was performed using JMP 15.0 software (JUMP, Cary, NC, USA). Significance tests were two-tailed and the results were considered significant at an α level of *p* < 0.05. Fischer’s tests were used to establish differences in the distribution of discontinuous variables. Student’s *t*-test or unidirectional ANOVA were used for continuous variables with a normal distribution, and Mann–Whitney U/Kruskal–Wallis tests were used to compare continuous variables without a normal distribution.

## 3. Results

### 3.1. Tumors and Patients

Our study included samples from 37 patients diagnosed with CM and 40 patients with conjunctival naevi (Table 1). These groups did not differ by gender, but a significant age difference was observed between patients with naevi (43.42 years, SD 3.32) and patients with conjunctival melanoma (67.89 years, SD 3.4) (Student’s *t*-test, *p* < 0.0001). Among the nevi, 25 nevi were compound and 15 subepithelial.

A total of 20 melanomas were located in the bulbar conjunctiva and 17 in the non-bulbar conjunctiva. 16 tumors belonged to the T1 category, 10 to the T2 category and 11 to the T3 category. Five melanomas arose from nevi, 25 from PAM and 7 de novo. The overall follow-up was 54.68 ± 10.64 months. Eight patients developed metastases and five patients died from the tumor. Recurrences were found in 69% of cases.

### 3.2. Expression of 5-hmC, 5-mC and TET2

**Figure 1:**
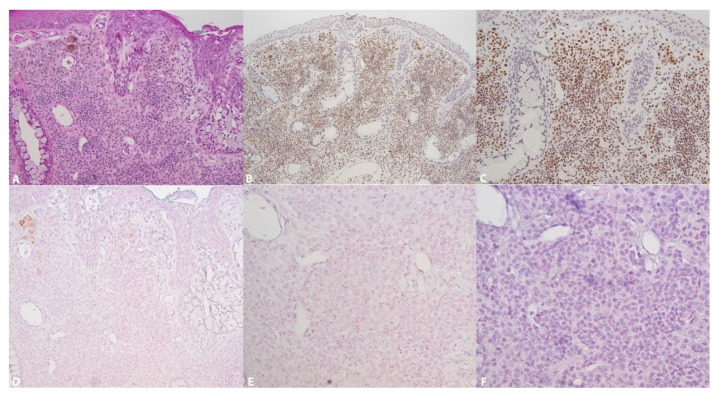
5-hmC and TET2 in a conjunctival nevus. (**A**): Subepithelial nevus (Hematoxylin-eosin, ×126); (**B**): Diffuse preservation of 5-hmC expression (×63); (**C**): Diffuse preservation at 5-hmC nuclei (×126); (**D**): Expression of TET2 by RNA ISH as red dots, score 2. TET2 is expressed in the conjunctival epithelium as an internal control (×126). (**E**): Enlargement of the lower part of D allowing the identification of several red dots/cell. Score 2 (×252). (**F**): In the same area, preservation of RNA integrity (POLAR2A) (×252).

**Figure 2:**
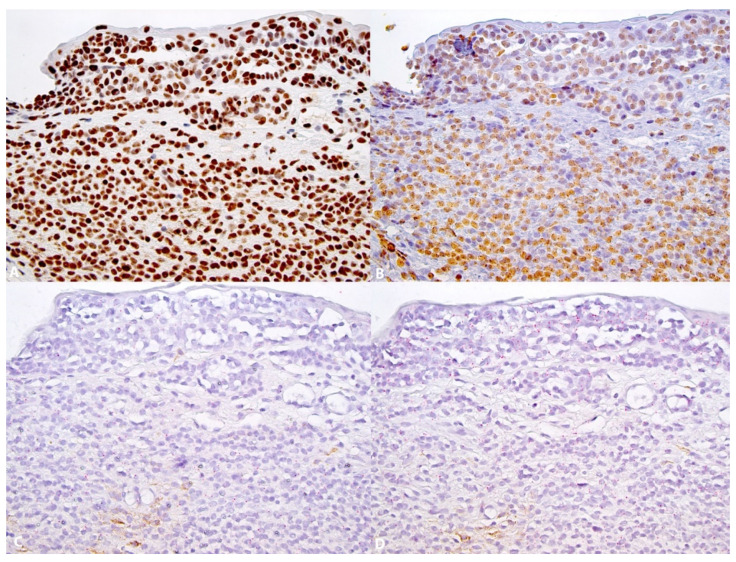
5-hmC, 5-mC and TET2 in a subepithelial conjunctival nevus. (**A**): Diffuse nuclear expression of 5-hmC (×252); (**B**): Diffuse preservation of 5-mC (×252). (**C**): Low expression of TET2, score 0–1 (×252); (**D**): In the same area, preservation of RNA integrity (POLAR2A) (×252).

**Figure 3:**
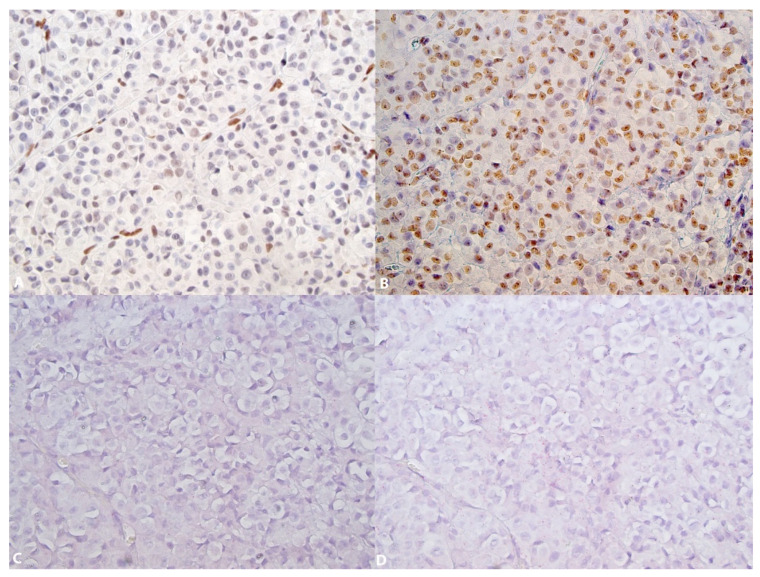
5-hmC, 5-mC and TET2 in a conjunctival melanoma. (**A**): Partial loss of 5-hmC expression. Strong nuclear 5-hmC expression can be seen in the endothelial cells (×252); (**B**): Preservation of 5-mC nuclear expression (×252); (**C**): Loss of TET2 expression (×252); (**D**): In the same area, preservation of RNA integrity (POLAR2A) (×252).

The expression of 5-hmC was identified in 100% of the nevi (Figure 1A (HE), Figure 1B,C and Figure 2A) and 54% of melanomas (Figure 3A) (Fischer’s test *p* > 0.0001) (Table 2). In the nevi, 5-hmC nuclear expression was diffusely found in the intraepithelial and subepithelial component and there was no loss of 5-hmC with maturation. In the melanomas, when 5-hmC was conserved, there was globally a diffuse expression. When 5-hmC expression was lost in these tumors, it was not possible to delineate specific areas for the occurrence of this loss, the loss being generally diffuse. Statistical analysis revealed a correlation between 5-hmC and local lymphatic invasion (Fischer’s test, *p* = 0.0383). 5-hmC loss was not associated with any other clinicopathological factors in melanomas (Table 3).

5-mC expression was identified in 67.5% of nevi (Figure 2B) and 81.105% of melanomas (Figure 3B) (Fischer’s test, *p* = 0.202) (Table 2). In both nevi and melanomas, 5-mC expression was globally diffuse and homogenous; it was not possible to identify areas with specific zonal loss of 5-mC. In melanomas, 5-mC expression was not associated with any clinicopathological factors (Table 3).

Both in conjunctival nevi and melanomas, the level of TET2 expression was low (score ranging from 0 to 1, rarely 2). TET2 expression was identified in 95% of nevi (Figure 1D–F (control) and Figure 2C,F (control)) and in 35.1% of melanomas (Figure 3C,D control) (Fischer’s test *p* > 0.0001) (Table 2). In the nevi, TET2 was homogeneously found in the intraepithelial and subepithelial components and TET2 expression was preserved with maturation. In the melanomas, TET2 loss was homogenously diffuse and it was not possible to identify localized areas with loss of TET2 expression. In melanomas, loss of TET2 was significantly associated with non-bulbar localization (*p* = 0.0423) and TNM classification (*p* = 0.0384), but not with other clinicopathological factors (Table 3).

Our study also identified a correlation between TET2 and 5-hmC expression (*p* = 0.0008), but there was no correlation between TET2 and 5-mC expression, nor was there a correlation between 5-mC and 5-hmC expressions.

## 4. Discussion

5-hmC loss is a distinctive epigenetic event in skin melanoma neoplastic progression that correlates with clinical relapse-free survival and melanoma staging parameters [13]. Lian CG et al. demonstrated reduced levels and distribution of 5-hmC at the melanoma epigenome compared to benign nevus [13]. Our results showed a similar significant reduction in 5-hmC expression in conjunctival melanomas compared to nevi. In addition to the study by Lian CG et al., 5-hmC expression has been evaluated in several studies in cutaneous melanocytic proliferations [15,16,17]. Our results are in line with these studies, which showed a significant loss of 5-hmC in melanomas compared to nevi [18]. In the study by Larson AR et al. [15], 5-hmC loss was evaluated by immunohistochemistry in a group of 175 cases including 18 benign cutaneous nevi, 20 dysplastic nevi, 10 atypical Spitz nevi, 20 borderline tumors, 5 cutaneous melanomas developing from nevi and 102 primary cutaneous melanomas. In this study, the authors observed a 5-hmC loss in dysplastic nevi and melanomas compared to nevi.

The level of 5-hmC in cells depends on several parameters, including TET and/or IDH enzymes, two key factors involved in the generation of 5-hmC. The functioning of TET enzymes as dioxygenases requires the use of α ketoglutarate as a co-factor [19]. This co-factor is produced by decarboxylation of isocitrate by IDH dehydrogenases. A decrease in TET2 or IDH1 reduced the generation of 5-hmC [13]. In our study, we focused on the level of TET2 detected by the RNA ISH technique: our results showed a significant decrease in TET2 in conjunctival melanomas compared to nevi. Our results are in line with those of another study [20] where significantly reduced levels of 5-hmC and TET2 in advanced melanomas compared to nevi and thin melanomas were reported. We preferred however the use of RNA in situ hybridization technology, as detection of TET2 by immunohistochemistry was not sufficiently convincing in our hands.

Ten percent of melanomas (4/39) harbor IDH1 or *IDH2* mutations [21], while no *TET* mutation has been reported in these tumors. The low penetration of *IDH* and *TET* mutations in skin melanoma suggests that other cancer pathways that inactivate these 5-hmC generating enzymes might play a major role in the negative regulation of 5-hmC [13]. However, in our study of the genomic and transcriptomic landscape of conjunctival melanoma [9], we identified IDH1 mutations in 29% of cases. It is therefore possible that the 5-hmC loss observed in conjunctival melanoma can also be partially attributed to IDH1 mutations.

As the level of 5-hmC partially depends on the level of 5-mC, we also determined the expression 5-mC in our tissues. We identified a non-significant increase of 5-mC level in melanomas compared to nevi. It is possible to speculate that this 5-mC increase in conjunctival melanomas might possibly be linked to decreased TET2 levels identified in our study. Our results in conjunctival melanoma differ however from those of previous studies in cutaneous melanoma where 5-mC level was significantly decreased in 97 melanomas comparing to 31 nevi in one study [22] and not significantly decreased in 61 melanomas comparing to 32 nevi in another study [20].

In skin melanoma, genome-wide analysis of the distribution of 5-mC and 5-hmC peaks showed a significant reduction of 5-hmC in melanomas in all regions of the genome compared to nevi, whereas there was only a slight reduction of 5-mC in melanomas compared to nevi [13]. In the same study, the analysis of areas where the level of 5-hmC was significantly higher than 5-mC in nevi and areas where the level of 5-mC was higher in melanomas compared to nevi led to the identification of more than 2000 genes involved in the Wnt signaling pathway or in adherent junctions. At the functional level, the authors of this study demonstrated in two different animal models that restoring the function of IDH1 or TET2 not only restored the level of 5-hmC in melanoma, but also reduced tumor growth.

In this study of 5-hmC, 5-mC and TET2 expression in tissues, we did not evaluate the distribution of 5-hmC and 5-mC which, combined with a study of the level of gene expression, would have allowed us to draw more precise conclusions on how the loss of 5-hmC influences gene expression in conjunctival melanoma. From a diagnostic perspective, however, the identification of 5-hmC loss in conjunctival melanomas remains a very valid element for the sometimes very difficult differential diagnosis between a melanoma and a conjunctival nevus. In this sense, the loss of 5-hmC in conjunctival melanoma could be an additional element to guide the clinical therapeutic attitude.

## 5. Conclusions

In conclusion, our study demonstrates a significant loss of 5-hmC in conjunctival melanoma compared to nevi, a loss not only linked to a decrease in the expression of TET2 but also possibly to alterations in its function as suggested by the presence of IDH1 mutations identified in 29% of conjunctival melanomas.

## Figures and Tables

**Table 1:** Tumors and clinical parameters.

Diagnostic	Total n	M	F	Middle Age (SD)	Tumor Subtype	*n*
Nevi	40	14	26	43.24 (23.54)	Compound nevus	25
					Subepithelial nevus	15
Melanoma	37	17	20	67.89 (17.95)	De novo	7 (18.9%)
					Pre-existing nevus	5 (13.5%)
					Pre-existing PAM	25 (67.57%)

M: male; F: female.

**Table 2:** Expression of 5-mC, 5hmC and TET2.

	5-mC	5-hmC	TET2
**Nevi *n* = 40**			
Score 0			2 (5%)
Score 1	13 (32.5%)		37 (92.5%)
Score 2	22 (55%)		1 (2.5%)
Score 3	5 (12.5%)	40	
**Melanomas *n* = 37**			
Score 0			24 (64.9%)
Score 1	7 (18.9%)	17 (46%)	13 (35.1%)
Score 2	16 (43.2%)	11 (29.7%)	
Score 3	14 (37.9%)	9 (24.3%)	

5-mC and 5-hmC: score 1 = positive staining in <10% of the cells; score 2 = positive staining in 10–50% of the cells; score 3 = positive staining in >50% of the cells. A score of 1 was interpreted as negative and scores of 2 and 3 as positive. TET2: Score 0 = no dots/cell; score 1 = 1–3 dots/cell; score 2 = 4–9 dots/cell; score 3 = 10–15 dots/cell; score 4 > 15 dots/cells).

**Table 3:** Clinicopathological correlations in conjunctival melanomas.

	5-hmC	5-hmC	*p*	5-mC	5-mC	*p*	TET2	TET2	*p*
	P	A		P	A		P	A	
**Age**									
>65	10	10		16	4		8	12	
<65	10	7	0.743 ^1^	14	3	1.0 ^1^	5	12	0.730 ^1^
**Sex**									
Male	11	6		15	2		5	12	
Female	9	11	0.324 ^1^	15	5	0.416 ^1^	8	12	0.731 ^1^
**Location**									
Bulbar	11	9		18	2		10	10	
Non-Bulb.	9	8	1.0 ^1^	12	5	0.212 ^1^	3	14	**0.0423 ^1^**
**Depth inv.**									
>0.5 mm	17			27	7		11	23	
<0.5 mm	3	17	0.234 ^1^	3	0	1.0 ^1^	2	1	0.277 ^1^
**Ki67**	23.7	35.9	0.0920 ^2^	29,1	33	0.572 ^2^	26.3	32.15	0.3668 ^2^
**Ly. Inv.**									
P	17	8		7	4		3	8	
A	3	9	**0.0383 ^1^**	23	3	0.1630 ^1^	10	16	0.7106 ^1^
**TNM**									
T1	9	7		14	2		9	7	
T2	7	3		9	1		3	7	
T3	4	7	0.2951 ^3^	7	4	0.209 ^3^	1	10	**0.0384 ^3^**
**Recurrence**									
P	12	13		21	4		7	18	
A	5	4	1.0 ^1^	6	3	0.3482 ^1^	5	4	0.224 ^1^
**Origin**									
Nevus	3	2		4	1		2	3	
PAM	14	11		21	4		6	19	
De novo	3	4		5	2		5	2	
**Death**									
P	3	3		4	2			6	
A	17	14	1.0 ^1^	26	5	0.315 ^1^	13	18	0.0719 ^1^

A: absent; P: present; Ly. Inv.: local lymphatic invasion; statistical tests: ^1^: Fischer’s; ^2^: Kruskall–Wallis; ^3^: Pearson. Significant correlations are in bold.

## Data Availability

The data presented in this study are available on request from the corresponding author.
